# Editorial: Exploring kidney pathology in transplantation: spotlight on non-neoplastic conditions and DCD donor quality

**DOI:** 10.3389/fmed.2026.1935998

**Published:** 2026-08-05

**Authors:** Alessandro Del Gobbo, Nadia Mansour, Alessandra Maria Storaci

**Affiliations:** 1 Division of Pathology, IRCCS Ca' Granda Foundation Maggiore Policlinico Hospital, Milan, Italy; 2 Department of Pathophysiology and Transplantation, University of Milan, Milan, Italy

**Keywords:** donation after brain and cardiac death, histology, kidney, pathology, transplant

Kidney transplantation remains the treatment of choice for patients with end-stage kidney disease, providing substantial benefits in terms of survival, quality of life, and social reintegration compared with long-term dialysis. Over recent decades, major progress in surgical techniques, perioperative management, immunosuppressive regimens, and donor-recipient matching has significantly improved short-term transplant outcomes. Nevertheless, important unmet needs persist. These include the accurate interpretation of allograft pathology, the early identification of patients at increased risk of perioperative and long-term complications, and the optimization of organ utilization in an era increasingly characterized by donor shortage and the use of more complex donor profiles.

In this context, kidney pathology occupies a central role. Histopathological assessment remains essential not only for the diagnosis of rejection and recurrent disease, but also for the evaluation of chronic injury, ischemic damage, donor-derived abnormalities, and the multiple non-neoplastic conditions that may influence graft function and long-term survival. At the same time, the expanding use of organs from extended-criteria donors and donors after circulatory death (DCD) has made the development of reliable diagnostic, prognostic, and predictive tools increasingly relevant. DCD kidneys, in particular, represent an important resource for expanding the donor pool, but their use is frequently associated with concerns related to ischemia-reperfusion injury, delayed graft function, and the challenge of accurately assessing organ quality before transplantation.

The Research Topic *Exploring kidney pathology in transplantation: spotlight on non-neoplastic conditions and DCD donor quality* was conceived as a platform for contributions addressing pathological, clinical, and prognostic aspects of transplantation medicine. Although the Research Topic does not focus exclusively on DCD donor pathology, the articles included share a common objective: improving risk stratification, diagnostic consistency, and clinical management across the transplant pathway. Together, they highlight the growing convergence of pathology, clinical medicine, digital technologies, and predictive analytics in the care of transplant recipients and donors.

A key challenge in kidney transplantation is the accurate and reproducible interpretation of allograft biopsies. Histopathology remains the cornerstone of diagnosing rejection and other transplant-related complications; however, the complexity of Banff classification criteria, the heterogeneity of pathological findings, and interobserver variability may represent significant obstacles to standardization. In this setting, Matsushita et al. evaluated the diagnostic performance of the Banff Automated Diagnosis System (ADS), a web-based tool designed to facilitate standardized classification of kidney transplant biopsies according to contemporary Banff criteria.

By analyzing more than one thousand biopsy results, the authors reported an overall concordance of approximately 70% between automated and expert pathologist diagnoses, with particularly high agreement for non-rejection and rejection categories. Importantly, discrepancies emerged in selected and clinically challenging settings, including antibody-mediated rejection in ABO-incompatible transplantation and chronic T-cell-mediated rejection. These observations underline both the potential and the current limitations of automated diagnostic systems. Digital tools may contribute to improving consistency, supporting training, and facilitating the application of standardized criteria across institutions; however, they cannot yet replace expert pathological interpretation, especially in complex cases requiring integration of morphology, immunopathology, clinical information, and transplant-specific context.

The study by Matsushita et al. is particularly relevant in the broader framework of digital pathology and artificial intelligence in transplantation. As whole-slide imaging, computational pathology, and decision-support systems become increasingly integrated into routine diagnostic workflows, their role will likely evolve from simple classification support toward more comprehensive platforms capable of incorporating histological, molecular, clinical, and longitudinal data. Such integration could be especially valuable in donor assessment and in the evaluation of marginal organs, where timely and reliable decisions are critical.

Beyond diagnostic pathology, the ability to anticipate clinical complications before they occur represents another major frontier in transplantation medicine. Dong et al. addressed this issue by investigating risk factors associated with intraoperative hyperkalemia during kidney transplantation and by developing a predictive nomogram model. Hyperkalemia remains a potentially life-threatening perioperative complication, with the potential to cause arrhythmias, hemodynamic instability, and adverse surgical outcomes. Identifying patients who are more likely to develop hyperkalemia may therefore support more proactive monitoring and earlier preventive interventions.

By integrating relevant clinical predictors into a practical risk assessment model, the authors provide an example of how data-driven approaches may enhance perioperative decision-making. Their work reflects the broader move toward personalized transplant medicine, in which clinical management is increasingly guided by individualized estimates of risk rather than by generalized protocols alone. In an increasingly complex transplant population, predictive models may help clinicians identify vulnerable patients, optimize resource allocation, and tailor perioperative strategies to specific donor-recipient combinations.

Long-term metabolic complications also remain important determinants of patient and graft survival. Post-transplant diabetes mellitus (PTDM) is among the most frequent and clinically relevant complications after kidney transplantation, contributing to cardiovascular morbidity, impaired graft function, infection risk, and reduced patient survival. Despite its clinical relevance, the optimal pharmacological management of PTDM remains an area in which direct comparative evidence is still relatively limited.

In their network meta-analysis, Hu and Lan compared the efficacy and safety of available antidiabetic therapies in patients with PTDM. Drawing on data from more than 7,000 patients, the authors found that insulin and sodium-glucose cotransporter-2 (SGLT2) inhibitors appeared to provide the most favorable balance between glycemic control and safety outcomes. Their findings contribute useful evidence to a clinically important and rapidly evolving field. In particular, the potential role of SGLT2 inhibitors in transplant recipients deserves continued attention, given their expanding use in the general population with diabetes and chronic kidney disease, alongside the need for careful evaluation of safety, drug interactions, infectious complications, and allograft-specific outcomes.

The Research Topic is further enriched by a study focused on predictive models for acute kidney injury in liver transplant recipients. Although this work is set within a different transplant context, it addresses a concept that is highly relevant across solid-organ transplantation: the early identification of patients at risk of organ dysfunction through robust and clinically applicable prediction tools. Acute kidney injury after transplantation remains a multifactorial complication, influenced by donor characteristics, recipient comorbidities, hemodynamic instability, ischemia-reperfusion injury, immunosuppressive exposure, and perioperative events. The development of reliable prediction models may therefore support earlier intervention and more individualized patient management.

Taken together, the contributions included in this Research Topic illustrate the increasing importance of multidisciplinary and integrative approaches in transplantation medicine. From automated interpretation of biopsy findings to perioperative risk prediction, evidence synthesis, and the management of post-transplant metabolic complications, these studies demonstrate how pathology and clinical medicine can be strengthened by digital tools, structured data, and advanced analytical methods.

Looking ahead, future research should continue to bridge conventional histopathological evaluation with molecular biomarkers, transcriptomic profiling, digital pathology platforms, and machine-learning algorithms. Such integration may be particularly valuable in the assessment of DCD donor kidneys, where the prediction of ischemia-reperfusion injury, delayed graft function, and long-term graft performance remains a central challenge. The development of reproducible, clinically validated tools capable of combining donor histology, clinical variables, preservation data, molecular information, and recipient characteristics may ultimately improve organ allocation and increase confidence in the use of kidneys that might otherwise be discarded.

At the same time, technological innovation should remain anchored to clinical relevance. Automated systems, predictive models, and artificial intelligence-based tools must be validated across diverse populations and transplant settings, and their implementation should complement rather than replace multidisciplinary clinical and pathological expertise. The most meaningful progress will likely arise from approaches that preserve the central role of expert interpretation while enhancing reproducibility, scalability, and precision.

Ultimately, improving transplant outcomes requires a more refined understanding of the complex interplay between donor quality, allograft pathology, perioperative events, recipient-specific risk factors, and long-term complications. The studies gathered in this Research Topic contribute to this evolving landscape by offering new perspectives on diagnostic standardization, individualized risk prediction, and evidence-based management. As transplantation medicine continues to move toward increasingly personalized and data-informed care, the integration of pathology, clinical expertise, and innovative analytical approaches will be essential to maximize graft utilization, improve patient outcomes, and support the continued development of precision transplantation.

